# Associations between cardiorespiratory fitness, fatness, hemodynamic characteristics, and sedentary behaviour in primary school-aged children

**DOI:** 10.1186/s13102-022-00411-7

**Published:** 2022-02-02

**Authors:** Garyfallia Pepera, Savvas Hadjiandrea, Ilias Iliadis, Gavin R. H. Sandercock, Ladislav Batalik

**Affiliations:** 1 Physiotherapy Department, Faculty of Health Sciences, University of Thessaly, 3rd km of Old National Road, 35100 Lamia, Greece; 2 School of Sport, Rehabilitation and Exercise Sciences, University of Essex, Wivenhoe Park, Colchester, Essex CO4 3SQ UK; 3 Department of Rehabilitation, University Hospital Brno, 62500 Brno, Czech Republic; 4 Department of Public Health, Faculty of Medicine, Masaryk University, 62500 Brno, Czech Republic

**Keywords:** Cardiorespiratory fitness, Obesity, Blood pressure, Hemodynamic, Fatness, Sedentary behaviour, Children

## Abstract

**Background:**

Low cardiorespiratory fitness (CRF) is associated with the development of cardiovascular diseases during childhood, adolescence and older ages. The purpose of the study was to investigate associations between fatness, hemodynamic characteristics and secondary time with CRF in primary school-aged children.

**Methods:**

Height, weight, body mass index (BMI), blood pressure (BP), heart rate (HR), CRF (20 m shuttle-run) and sedentary time were measured for 105 children (categorized as normal, overweight, obese). The independent sample t-test checked for differences and one-way ANOVA—Post Hoc Test and stepwise linear regression analysis assessed the 20 m shuttle-run performance predictors.

**Results:**

There was a statistically significant difference in CRF between boys and girls. There was a statistically significant difference between (*p* < 0.05) CRF for Normal weight (M = 47.58 ± 3.26 kg m^−2^) and Obese (M = 44.78 ± 3.23 kg m^−2^). CRF correlated with age, BMI and sedentary time (r > 0.3; *p* < 0.05). BMI is the best independent predictor of CRF.

**Conclusions:**

Children with normal BMI tend to present better CRF performance than obese and overweight children. Sedentary behaviour is associated with lower CRF in primary school-aged children.

## Background

Cardiorespiratory fitness (CRF) is an objective indicator of physical activity and a useful prognostic and diagnostic capacity tool [1]. It is directly related to the cardiovascular system, respiratory system, musculoskeletal system and is widely considered the best indicator of health status [2]. Low CRF is associated with the development of cardiovascular diseases during childhood, adolescence [3] and older ages [4]. High levels of CRF are associated with a good health index, [5] and low levels with various cardiovascular diseases such as hypertension and type 2 diabetes [6–8]. Physical activities and increased CRF positively affect each of the different components of metabolic syndromes.

Being overweight has a negative impact on children’s CRF and overall performance [9]. Poor CRF, obesity and fatness appear as serious risk factors for various cardiovascular diseases, early stages of atherosclerosis and obesity in children and adolescents [10]. Moreover, a sedentary lifestyle based on self-reported sedentary behaviour has been identified as a risk factor, with a detrimental association to cardiovascular diseases [11].

Many parameters (anthropometric, hemodynamics, quality of life) can affect or even be affected by CRF [12–14]. Surprisingly, research has not yet to establish the relation considering fatness, hemodymanic characteristics and sedentary behaviour together in CRF for Greek primary school-aged children. Given that the results of the measurements indicate clear evidence that reduced CRF tends to be associated with the development of cardiovascular disease, the aims of this study were fourfold. The main aims were to determine the associations between fatness, hemodynamic characteristics and secondary time with CRF in primary school-aged children. Second, we sought to discover the best predictor of CRF performance in this population.

## Methods

### Study design

The research study was approved by the Ethics Committee of the University of Thessaly (308ΣΕ2/27-01-2020) stating that the research proposal is in accordance to the international principles of ethical practice and ethics which are in line with the value of respect for the volunteers who will participate. The measurements of the research were carried out in Greece and included, measurement of CRF, anthropometric characteristics (height, weight, BMI), hemodynamic characteristics (systolic blood pressure; SBP, diastolic blood pressure; DBP, heart rate; HR), and sedentary behaviour (via the HELENA questionnaire).

### Study population

A convenience sample of 105 children aged 6–12 years participated. The sample size was calculated according to a sample size calculation algorithm (sample size calculator) with confidence level = 95% and confidence interval = 0.05. There were 141 children in total in the academy, so the algorithm estimated that 103 children were needed for the sample of the present study. Participants gave informed parental consent to participate in this study. All children normally participated in academy training were included in the study; exclusion criteria were children of potential illness and lack of consent.

### Measurements protocol

The measurements were conducted during a scheduled training session. Each participant or caregiver received an information form for the measurements that followed, a written consent form, and the HELENA questionnaire regarding the time spent watching the screen on electronic devices.

**Fig. 1:**
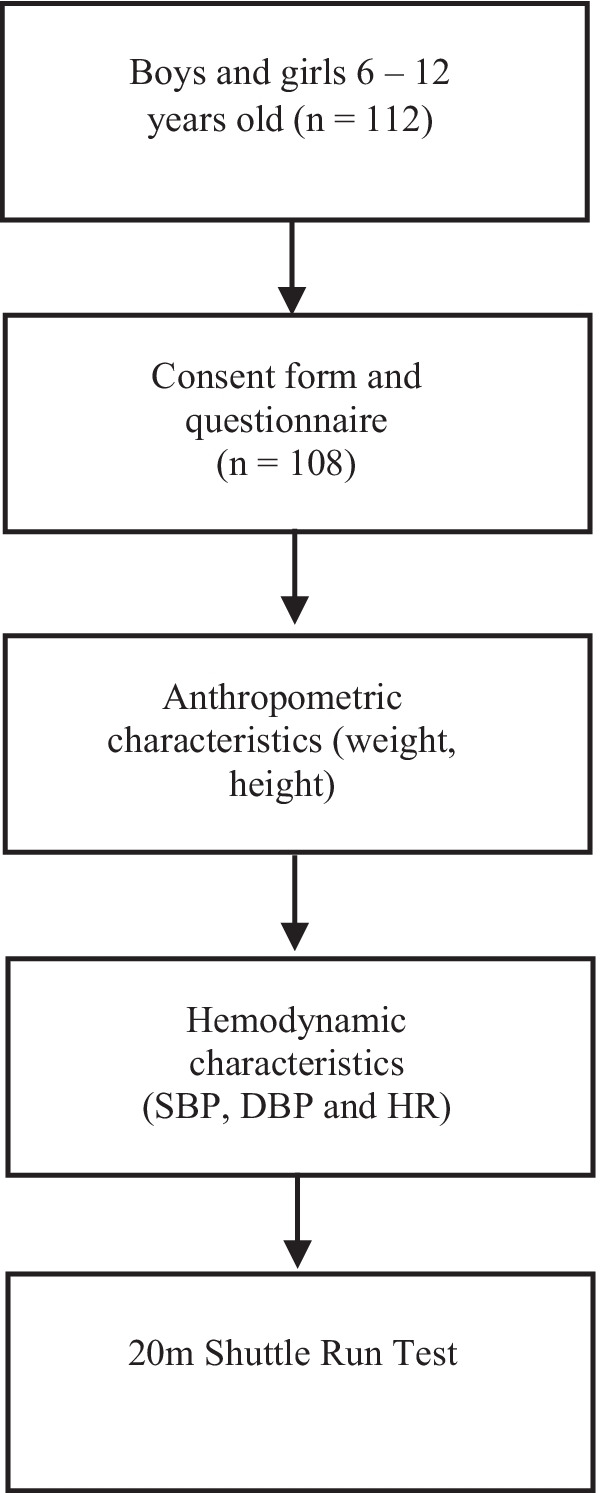
Study flow chart. The figure shows a flowchart of the study

Only subjects who returned the consent form signed and the questionnaire completed by parents or by themselves, participated in the study. All assessments were performed between 3 and 7 p.m. Most of the measurements (anthropometrics, grip strength, hemodynamic) were conducted before training. First, the anthropometric parameters (height, weight) were measured and the hemodynamic characteristics were recorded (SBP, DBP, HR) and the measurement of the CRF (20 m shuttle run test) followed (Fig. 1).

### Anthropometric measurements

Anthropometric measurements included stature and weight. During anthropometric measurements, participants wore light wear and barefoot. Stature was measured to the nearest millimetre using a stadiometer and weight was measured to the nearest 0.1. kg [15] using a reliable weighing scale (TANITA BC418-MA) [16]. BMI was calculated based on the International System of Units, BMI = weight (kg)/height^2^ (m^2^) and expressed as z-score in the categories NORMAL, OBESE and OVERWEIGHT [17].

### Hemodynamic characteristics

Systolic Blood Pressure (SBP), Diastolic Blood Pressure (DBP), and Heart Rate (HR) were measured before the 20 m Shuttle Run Test (20-mSRT). Blood pressure (BP) and HR were measured with the Microlife BP A2 Basic automated brachial sphygmomanometer [18] using sized cuff for children (cuff perimeter of 17–22 cm). Before the measurement, the children took off any cardigans and jackets they were wearing to decompress the arm area as much as possible so as not to affect the measurement. The cuff was placed on the left arm of each child and specifically on the brachial artery at the height of the heart.

Children were requested to remain relaxed in a sitting position for at least 10 min prior to the measurement. The selected position for the hemodynamic measurement was A total of two measurements were conducted for each participant, taking into account the lowest value obtained for SBP and DBP [19], while the mean of HR measurements of each participant was used in the analysis [20]. During the BP and HR recording, the participant was asked to sit upright with legs resting on the ground without being crossed and both elbows resting on the table surface where the measurement was being made.

The categorization of SBP and DBP of the sample was done according to the norms of the American Academy of Paediatrics because BP differs according to age and gender [21]. Each participant was categorized according to age and height, in the following categories: ‘Low’, ‘Normal’, ‘High’ for both boys and girls. Finally, norms were used [22] to classify each participant according to age and the average value of the HR in the following categories: ‘Below normal’ and ‘Above normal’.

### Cardiorespiratory fitness

CRF was assessed using a reliable, valid and fissile cardiorespiratory test [23] the 20-mSRT, based on the prototype of Leger [24]. During the 20-mSRT, the standard procedure was followed, and one preliminary attempt was performed. The 20-mSRT was administered using the BeepShuttle Advanced VER0320 program. This test was performed by running continuously between two points at a distance of 20 m and the time of change from one point to another was determined by audio feedback with the characteristic ‘beep’ sound [25]. Children ran to an audible signal pace at an initial speed 8.5 km h^−1^ and speed was increased by 0.25 km h^−1^ every minute. After each minute, a vibrating sound indicated an increase in speed level (level change) [25]. Test termination criteria were: a. when the participant was unable to continue the test because of fatigue or other symptoms (voluntary withdrawal), b. when the participant failed to reach the marker on time, c. completion of all levels [26].

At the end of the 20-mSRT, performance was recorded as the number of shuttles each participant completed, which was then converted to the final running speed (km h^−1^) at the final completed stage [25]. This software implemented the standard one-minute protocol which started at a speed of 8.5 km h^−1^ and increased by 0.5 km h^−1^ after each minute. Maximum oxygen uptake (VO_2_max) was calculated indirectly by the equation: VO_2_max (ml kg^−1^ min^−1^) = 31.025 + (3.238 × speed) − (3.248 × age) + (0.1536 × speed × age).

The scores were categorized as follows: Very Poor (< 10%), Poor (10–25%), Moderate (25–75%), Good (75–90%) and Excellent (≥ 90%) [25].

### Sedentary time

The time of sedentary behaviour was assessed by a self-administered questionnaire HELENA. Participants were asked to report the usual time devoted to several sedentary behaviours during weekdays and weekends. The categories were: TV show, computer games, console (video) games, Internet for non-school obligations or other reasons (hobbies), Internet for educational purposes and study time (outside the study program). Participants had to choose between one of the followings: 0 min, > 0–30 min, > 30–60 min, > 60–120 min, > 120–180 min, > 180–240 min and > 240 min for each of the 12 questions.

Total weekday and weekend sedentary time was calculated by summing the time spent on each activity. The weekly time was calculated by taking the average time in the selected category and applying this formula: [(daily × 5) + (weekend × 2)]/7. The sitting minutes per day were calculated as follows: category 1 = 0 min, 2 = 15 min, 3 = 45 min, 4 = 90 min, 5 = 150 min, 6 = 210 min and 7 = 241 min, respectively [27].

### Statistical analysis

Anthropometric factors (weight, height, BMI), hemodynamic parameters (SBP, DBP, HR) as well as sedentary time were defined as independent variables. The maximum CRF was defined as the dependent variable.

Descriptive statistics were performed to derive results on the mean of the variables (mean) and the standard deviation (SD). At the same time, regularity was checked with Kolmogorov–Smirnov in SPSS. The independent sample t-test was used to identify possible differences in the dependent and independent variables between the two genders (*p* < 0.05) with a significance level of 95%. Analysis of Variance (ANOVA Post-Hoc Test) was performed between the three groups (Normal, Overweight, Obese) based on the z-score for BMI and VO_2_max. Stepwise linear regression analysis was used to find a model for assessing CRF (VO_2_max) based on the values of age, BMI and sedentary time. The statistical significance of the Total Regression Model (ANOVA) was then tested with the Dispersion Analysis Table (ANOVA). The ratio of the coefficients to the dependent variable (VO_2_max) was calculated, from which the prediction equation for VO_2_max was created. The statistical analysis of the survey data was performed using the SPSS version 25.0 (SPSS Inc, Chicago, IL, USA.). Statistical significance was set on < 0.05. Cohen’s d measure was based on the difference between three means: small (d = 0 to 0.2), medium (d = 0.3 to 0.5) and large (d ≥ 0.6).

## Results

Descriptive characteristics are presented in Table 1. A total of 105 children participated in the study, where 84% of the participants were boys. Mean values for age was 10.52 ± 1.94 years and for BMI was 19.97 ± 3.47 kg m^−2^. According to the BMI 32% of the children were obese (> 30 kg m^−2^), 23% were overweight (25–29.99 kg m^−2^) and 45% were normal (< 25 kg m^−2^). The Kolmogorov–Smirnov method was performed to examine the level of normality of the cardiorespiratory variables (*p* value > 0.05).

**Table 1:** Descriptive statistics

	Age (years)		Height (m)		Weight (kg)		BMI (kg m^−2^)	
	Mean	SD	Mean	SD	Mean	SD	Mean	SD
Total N = 105	10.52	1.94	1.45	0.12	41.97	12.22	19.97	3.47
Boys N = 88	10.41	1.95	1.43	0.12	41.49	12.10	19.86	3.44
Girls N = 17	11.12	1.80	1.46	0.15	44.43	12.93	20.50	3.64

*SD* standard deviation, *BMI* body mass index

Specifically, CRF was higher in boys (46.8 ± 3.4 ml kg^−1^ min^−1^) and girls (45.1 ± 1.9 ml kg^−1^ min^−1^) (Table 2). The mean difference of 1.6 ml kg^−1^ min^−1^ (95% CI 0.43 to 2.8) indicates a moderate effect size d = 0.58. The one-way ANOVA—Post Hoc Test was performed for analysis of variance between groups and to find the influence of BMI on CRF. CRF showed a statistically significant difference (*p* < 0.05) between normal weight and obese children: F (2,103) = 6.59, *p* = 0.002. Post-Hoc comparisons using Tukey HSD indicated that the mean score of CRF for group 1 (M = 47.58 ± 3.26 ml kg^−1^ min^−1^) was statistically significant different from the average score of Group 3 (M ± 3.23 ml kg^−1^ min^−1^). In contrast, the mean score of Group 2 (M = 46.18 ± 2.86 ml kg^−1^ min^−1^) was not statistically significantly different from any of the other groups (Tables 2, 3).

**Table 2:** Distribution of groups based on cardiorespiratory fitness (VO_2_max)

	Group	Means difference	SE difference	SE	Mean difference 95% CI	Min	Max
*Cardiorespiratory fitness, VO*_*2*_*max* (ml kg^−1^ min^−1^)	NORMAL < 25 kg m^−2^	47.58	3.26	0.480	47.57 (46.60–48.54)	41.1	54.0
	OVERWEIGHT 25–29.99 kg m^−2^	46.18	2.86	0.490	46.19 (45.19–47.18)	40.5	52.2
	OBESE > 30 kg m^−2^	44.78	3.23	0.660	44.78 (43.42–46.14)	39.5	54.4

*VO*_*2*_*max* cardiorespiratory fitness

**Table 3:** Comparisons between groups and cardiorespiratory fitness

BMI groups (z-score)	Mean	SD	*p* value	Mean difference 95% CI
VO_2_max (normal–overweight)	1.4	0.7	0.121	3.351 (− 0.277 to 3.074)
VO_2_max (normal–obese)	2.8	0.78	0.002	5.595 (0.930 to 4.665)
VO_2_max (overweight–obese)	1.4	0.83	0.219	3.968 (− 0.585 to 3.383)

*BMI* body mass index, *VO*_*2*_*max* cardiorespiratory fitness, *SD* standard deviation

A correlation was made between dependent and independent variables as shown in Table 4. There was a statistically significant negative correlation between age (r = − 0.398) (*p* < 0.05), height (r = − 0.287) (*p* < 0.05), weight (r = − 0.463) (*p* < 0.05), and BMI (z-score) (r = 0.338) (*p* < 0.05) with CRF. The same results were observed in the hemodynamic characteristics where there was a negative statistically significant correlation for SBP (r = − 0183) (*p* < 0.05), DBP (r = − 0.205) (*p* < 0.05) and HR (r = − 0.159) (*p* < 0.05). Finally, in sedentary behaviour, there was also a statistically significant negative correlation with sedentary time (r = − 0.313) (*p* < 0.05).

**Table 4:** Correlation of the independent with the dependent variable

	Cardiorespiratory fitness (ml kg^−1^ min^−1^)	
	r	*p* value
Age (years)	− 0.398	< 0.005
Height (m)	− 0.287	0.003
Weight (kg)	− 0.463	< 0.005
BMI (kg m^−2^)	− 0.504	< 0.005
BMI (z-score)	− 0.338	< 0.005
SBP (mmHg)	− 0.183	0.062
DBP (mmHg)	− 0.205	0.036
HR (bpm)	− 0.159	0.104
ST (min)	− 0.313	0.006

*BMI* body mass index, *SBP* systolic blood pressure, *DBP* diastolic blood pressure, *HR* hart rate, *ST* sedentary time

A stepwise linear regression analysis was used to assess the combined associations. For the regression analysis, only the independent variables were used. The independent variables selected for the analysis have been found to be statistically significant (*p* < 0.05) and have a correlation of r > 0.3 with CRF. The correlation coefficient (r) with the other independent variables was less than 0.75 to avoid the high correlation between the variables (Meyers et al. 2006, p. 366). The regression analysis showed that age and BMI should be included in the final model. In this model, 31.6% of CRF was explained by age and BMI with a standard error of 1.969 ml kg^−1^ min^−1^ (Table 5). Predicted CRF would be computed by using the equation form for each individual: CRF (VO_2_max) = 59.429 − (0.395 × BMI) − (0.479 × age), in ml kg^−1^ min^−1^.

**Table 5:** Predictors of maximum cardiorespiratory fitness (VO_2_max)

	B	SE	Beta	t	*p* value	95% CI for B
Age	− 0.479	0.152	− 0.256	− 3.155	0.002	− 0.780 to − 0.178
BMI (kg m^−2^)	− 0.395	0.085	− 0.408	− 4.665	< 0.005	− 0.564 to − 0.227
Constant	59.429	1.969	–	29.703	< 0.005	55.520 to 63.338

*BMI* body mass index, *SE* standard error

## Discussion

Τhis study determined that CRF is significantly negatively correlated with both fatness and hemodynamic characteristics as well as sedentary behaviour. Regarding the age of the children, there is a statistically significant negative correlation with the CRF. As children grow older, CRF decreases. Τhe results of a similar study showed to some extent, that in the age range of 12–19 years, boys had higher rates of CRF at older ages while girls at younger ages [28].

### Association between fatness and cardiorespiratory fitness

Regarding body weight, a statistically significant negative correlation was found with CRF. This finding is in agreement with other studies [29, 30]. The correlation between children's height and CRF was statistically negatively significant, in contrast to what other studies suggest [31, 32]. Α possible explanation for this difference could be the age range used in this study.

The present results showed a statistically significant negative correlation between BMI variables and CRF. Specifically, children in the 'normal' (BMI < 25 kg m^−2^) and 'overweight' (BMI from 25 to 29.99 kg m^−2^) categories had higher rates of CRF than children in the category 'obese' (BMI > 30 kg m^−2^). This is in line with the findings of Aires et al. (2010) while Mota et al. (2006) showed correlation in girls only [33, 34]. The prevention and treatment of obesity need more emphasis since obese and overweight children are more likely to remain obese into adulthood as opposed to children who have normal weight. It is also more difficult to lose excess weight as an adult compared to younger ages [35].

No gender comparison was performed because there is a difference between the number of boys and girls and also because anthropometric characteristics, differences for body build measures associated with musculoskeletal size, muscularity, skeletal size, total body mass, or body breadth dimensions of these ages are not different.

### Association between hemodynamic characteristics and cardiorespiratory fitness

Regarding hemodynamic characteristics, and in particular SBP, there was a statistically significant negative correlation with CRF. This is in agreement with Agostinis-Sobrinho et al. (2018) where in a sample of adolescents while an older study of Heggebø et al. (2006) claimed the opposite as they showed a positive correlation in a greater sample of 4072 children between 9–15 years old [34, 36]. The mechanism that may explain the negative correlation found is not yet clear, however, it is known that CRF can be modified with exercise and can prevent increased BP through changes in insulin sensitivity, endothelial function and function of the autonomic nervous system [34]. CRF increase positively affects BP since it is related to lower vascular peripheral resistance and activity of the sympathetic nervous system, reduced hormones such as noradrenaline and increased lumen diameter and length and mucle and adipocytes [37].

Regarding resting HR, there was a negative correlation with CRF. The same result was declared by Silva et al. (2018) indicating that the improvement of CRF is closely related to an increased left ventricular diameter and ultimately to an increased systolic volume [20]. Additionally, HR in relation to CRF can affect the autonomic nervous system. In particular, there is a decrease in circulating catecholamine levels and changes in the number or affinity of receptors [38]. Similarly, the same results were obtained by the research of Kang et al. (2017) with the difference that the sample was older. HR assessment is a very important parameter for general health and especially in metabolic and cardiovascular diseases [39]. Particularly normal resting HR values appear to help reduce the risk of developing dyslipidemia in obese children and adolescents [40].

According to a review when CRF is a marker of VO_2_max per kg body mass, then there is an opposing influence of obesity. Negative correlations between CRF, body mass and body fat measures are high (r = − 0.50 to − 0.80). This was explained as a result of inert adipose tissue, which inflates the denominator (“per kg”) and lowers mass-adjusted maximal aerobic power [41]. A study found higher absolute VO_2_max in children that are obese compared non-obese children during treadmill exercise (1.56 ± 0.40 vs 1.24 ± 0.27 l min^−1^) however the difference disappeared when VO_2_max was calculated according to fat free mass (59.2 ± 4.9 and 57.9 ± 5.8 ml kg^−1^) [42]. Therefore, the negative correlation found in this study between VO_2_max per kg and the level of obesity may not signify any cardiac issue and more studies are needed to evaluate this.

### Association between sedentary behaviour and cardiorespiratory fitness

Regarding the sedentary time, there was a statistically significant negative correlation with the CRF. Children with increased sedentary lifestyles tended to have lower levels of CRF. This is in agreement with the findings of Sandercock et al. (2016) that claim that higher sedentary time associated with an increased likelihood of decreased CRF [4].This indicates a higher fat mass that has a negative impact on the health of the population, as it is associated with an increased likelihood of developing various metabolic syndromes [43].

Sedentary time is a health detrimental factor that promotes physical inactivity. Many ways of managing reduced physical activity have been reported which contribute closely to cardiorespiratory levels. Respectively with the CRF, physical exercise is negatively related to the time of exposure to electronic devices (screen time) [44]. However, some hints can be used as an effective short-term way to reduce screen time. Educating parents about the dangers of prolonged use of these devices can also be an effective time-saving measure [44]. Also, the way children go to school can affect CRF. In particular, children who went to school on foot or by bicycle had higher rates of CRF as well as a reduced risk of developing cardiovascular disease [15].

### Which is the best predictor of shuttle run test performance?

Regression analysis showed that BMI was the most important predictive value for CRF, but that age was also significantly correlated with CRF in primary school-aged children. Sedentary time is a changeable characteristic, which cannot be easily measured in clinical practice, especially amongst the children. The use of BMI and age as the predictor of CRF in children has utility, because it is easy to measure. These findings indicate that the interpretation of CRF is not accurate without taking age in account. It’s obvious that younger children have an advantage over older children (6–12 years old) when performing a CRF test. There is a great deal of variation in the international literature on the prediction variables i.e. gender, BMI, HR, physical activity used to determine the CRF in children [26, 45].

### Limitations

The initial limitation of the study was the sample composition since there was a significant difference in the percentage of the two genders participating in this study (88 boys and 17 girls). This may affect the generalization of the results in the population. Also, in the process of measurements, there were some factors that more or less influenced some of the tests. More specifically, some children had already been 'warmed up' before they even came to training, while others did not. Additionally, some children had eaten before training since the training time was almost immediately after school. Regarding the questionnaire, because it was filled out by the parents, there may have been parents whose answers did not correspond to reality.

### Future research

Future research can investigate the correlations that exist in the Greek population and other nationalities with different socioeconomic statuses. Research could also be conducted to update norms on anthropometric, hemodynamic characteristics and CRF. Finally, regarding the interaction of CRF and obesity, it is suggested that more longitudinal studies should be performed taking gender into account to investigate this correlation in the long run since the rates of obesity are upward.

## Conclusion

The above results are of high clinical importance and add new findings to the literature with special significance in clinical practice. These data show that children with a normal BMI have better CRF than obese and overweight (fatness) children. Besides, increased cardiorespiratory values appeared to be associated with lower SBP, DBP, and HR values. Still, the reduced sedentary time was shown to be related to high CRF.

The findings of the present study are important with regard to showing that younger and normal-weight children have an advantage in the CRF performance and it should be accounted for age and BMI when interpreting 20-mSRT. The reference equation generated here shows that age and BMI should be accounted for in estimating CRF in primary school-aged children. By interpreting CRF as a percentage of the predicted value, practitioners would gain a more meaningful assessment of individual children’s CRF. Adding this simple predictor (age) to the standard CRF assessment may help clinicians provide more realistic CRF improvement targets.

All these correlations that emerged are a guide to good health. Maintaining these variables at satisfactory levels from an early age reduces the likelihood of developing cardiovascular disease during childhood and adolescence and provides a better cardiovascular profile. In conclusion, reducing sedentary behaviour, reducing screen leisure time, encouraging physical activity, promoting a healthy lifestyle with the pillar of maintaining CRF at high values from childhood, could provide a better cardiovascular profile and reduce the risk of developing cardiovascular disease in adulthood.

## Data Availability

Data are however available from the authors upon reasonable request and with permission of corresponding author.

## Abbreviations

CRF: Cardiorespiratory fitness
BMI: Body mass index
HR: Heart rate
SBP: Systolic blood pressure
DBP: Diastolic blood pressure
BP: Blood pressure
20-mSRT: 20 M shuttle run test
SD: Standard deviation
